# Signatures of transmission in within-host *Mycobacterium tuberculosis* complex variation: a retrospective genomic epidemiology study

**DOI:** 10.1016/j.lanmic.2024.06.003

**Published:** 2024-11-28

**Authors:** Katharine S Walter, Ted Cohen, Barun Mathema, Caroline Colijn, Benjamin Sobkowiak, Iñaki Comas, Galo A Goig, Julio Croda, Jason R Andrews

**Affiliations:** Division of Epidemiology, University of Utah, Salt Lake City, UT, USA; Department of Epidemiology of Microbial Diseases, Yale School of Public Health, New Haven, CT, USA; Department of Epidemiology, Columbia University Mailman School of Public Health, New York, NY, USA; Department of Mathematics, Simon Fraser University, Burnaby, BC, Canada; Department of Epidemiology of Microbial Diseases, Yale School of Public Health, New Haven, CT, USA; Institute of Biomedicine of Valencia, Valencia, Spain; Swiss Tropical and Public Health Institute, Allschwil, Switzerland; University of Basel, Basel, Switzerland; Department of Epidemiology of Microbial Diseases, Yale School of Public Health, New Haven, CT, USA; Federal University of Mato Grosso do Sul, Campo Grande, Brazil; Oswaldo Cruz Foundation Mato Grosso do Sul, Campo Grande, Brazil; Division of Infectious Diseases and Geographic Medicine, Stanford University School of Medicine, Stanford, CA, USA

## Abstract

**Background:**

*Mycobacterium tuberculosis* complex (MTBC) species evolve slowly, so isolates from individuals linked in transmission often have identical or nearly identical genomes, making it difficult to reconstruct transmission chains. Finding additional sources of shared MTBC variation could help overcome this problem. Previous studies have reported MTBC diversity within infected individuals; however, whether within-host variation improves transmission inferences remains unclear. Here, we aimed to quantify within-host MTBC variation and assess whether such information improves transmission inferences.

**Methods:**

We conducted a retrospective genomic epidemiology study in which we reanalysed publicly available sequence data from household transmission studies published in PubMed from database inception until Jan 31, 2024, for which both genomic and epidemiological contact data were available, using household membership as a proxy for transmission linkage. We quantified minority variants (ie, positions with two or more alleles each supported by at least five-fold coverage and with a minor allele frequency of 1% or more) outside of PE and PPE genes, within individual samples and shared across samples. We used receiver operator characteristic (ROC) curves to compare the performance of a general linear model for household membership that included shared minority variants and one that included only fixed genetic differences.

**Findings:**

We identified three MTBC household transmission studies with publicly available whole-genome sequencing data and epidemiological linkages: a household transmission study in Vitória, Brazil (Colangeli et al), a retrospective population-based study of paediatric tuberculosis in British Columbia, Canada (Guthrie et al), and a retrospective population-based study in Oxfordshire, England (Walker et al). We found moderate levels of minority variation present in MTBC sequence data from cultured isolates that varied significantly across studies: mean 168·6 minority variants (95% CI 151·4–185·9) for the Colangeli et al dataset, 5·8 (1·5–10·2) for Guthrie et al (p<0·0001, Wilcoxon rank sum test, *vs* Colangeli et al), and 7·1 (2·4–11·9) for Walker et al (p<0·0001, Wilcoxon rank sum test, *vs* Colangeli et al). Isolates from household pairs shared more minority variants than did randomly selected pairs of isolates: mean 97·7 shared minority variants (79·1–116·3) versus 9·8 (8·6–11·0) in Colangeli et al, 0·8 (0·1–1·5) versus 0·2 (0·1–0·2) in Guthrie et al, and 0·7 (0·1–1·3) versus 0·2 (0·2–0·2) in Walker et al (all p<0·0001, Wilcoxon rank sum test). Shared within-host variation was significantly associated with household membership (odds ratio 1·51 [95% CI 1·30–1·71], p<0·0001), for one standard deviation increase in shared minority variants. Models that included shared within-host variation versus models without within-host variation improved the accuracy of predicting household membership in all three studies: area under the ROC curve 0·95 versus 0·92 for the Colangeli et al study, 0·99 versus 0·95 for the Guthrie et al study, and 0·93 versus 0·91 for the Walker et al study.

**Interpretation:**

Within-host MTBC variation persists through culture of sputum and could enhance the resolution of transmission inferences. The substantial differences in minority variation recovered across studies highlight the need to optimise approaches to recover and incorporate within-host variation into automated phylogenetic and transmission inference.

**Funding:**

National Institutes of Health.

## Introduction

Reducing the global burden of tuberculosis urgently requires reducing the number of incident *Mycobacterium tuberculosis* complex (MTBC) infections. Yet the long and variable latency period of these infections makes it challenging to identify sources of transmission and thus intervene. Genomic epidemiology approaches have been powerfully applied to characterise MTBC global phylogenetic structure, migration and gene flow, patterns of antibiotic resistance, and transmission linkages.^1^ Yet transmission inference approaches have often failed to identify the majority of transmission linkages in high-incidence settings.^2,3^ Further, although previous studies have identified heterogeneity in the number of secondary cases generated by infectious individuals^4^ and risk factors for onward transmission,^5,6^ these are often difficult to generalise. Many crucial questions, including the contribution of asymptomatic individuals to transmission, remain unanswered. Novel, accessible approaches to reconstruct high-resolution transmission patterns are urgently needed so that public health programmes can identify environments driving transmission and risk factors for onward transmission.

Commonly used approaches for MTBC transmission inference use single consensus genomes, representing the sequence of the most frequent alleles, from infected individuals. Closely related pathogen genomes are predicted to be more closely linked in transmission chains. For example, closely related MTBC consensus sequences, with a pairwise genetic distance under a given threshold, are considered clustered and potentially epidemiologically linked.^7^ However, MTBC evolves at a relatively slow rate.^8^ The result is that there might be limited diversity in outbreaks. Several genomic epidemiology studies reported that multiple individuals harboured identical MTBC genomes, making it difficult to reconstruct who infected who.^9^ This challenge highlights a need to recover more informative variation from pathogen genomes, a challenge not unique to MTBC.^10^

Population-level bacterial diversity within an individual, or within-host heterogeneity, can be attributed to mixed infections (ie, infections with more than one distinct MTBC genotype) or de novo evolution (ie, mutations that are introduced over the course of an individual’s infection).^11^ Previous research has found that a substantial proportion (10–20%)^11^ of infected individuals harbour mixed infections with genetically diverse populations of MTBC.^11,12^ A portion of within-host heterogeneity is probably transmitted onward^13^ and therefore, within-host diversity captures potentially valuable epidemiological information about transmission history.^13^ Complex infections are also important clinically. Within-host heterogeneity is associated with poor treatment outcomes,^12,14^ and heteroresistance—presence of bacteria cells exhibiting different levels of susceptibility to specific antibiotics—reduces the accuracy of diagnostics for antibiotic resistance.^14^

Given that minority variation is frequently observed, we might expect that it could improve resolution of transmission and phylogenetic inference. Yet there are many open questions about whether shared within-host variation is a predictor of transmission linkage and, more practically, how to recover this level of variation and incorporate it into transmission inferences. Currently, MTBC is most frequently cultured from sputum samples and sequenced with short reads to generate a single consensus sequence.^1^ First, this approach limits the variation recovered because culture imposes a severe bottleneck, because there might be small numbers of cells from minority populations in sampled sputum, competition or stochastic growth in culture might result in loss of minority variants, and cultured samples are often subdivided for sequencing.^15,16^ Second, within-host variation, including mixed infections, is often excluded, in part due to an absence of validated methodological approaches for accurate recovery of such variation.^13,16^ Third, repetitive genomic regions, including the PE and PPE gene families, among the most variant-rich and potentially informative regions of the genome, are excluded.^17-19^

MTBC transmission is never directly observed, and in practice, epidemiological linkages are frequently unknown. This unknown makes it difficult to assess the performance of genomic methods in identifying true transmission linkages. We therefore aimed to leverage previously published household transmission studies to test whether household members—as a proxy for epidemiologically linked individuals—shared more minority variants than did unlinked individuals. We then aimed to test whether shared minority MTBC variation might augment fixed genomic differences in reconstructing epidemiological linkages and might enhance transmission inferences.

## Methods

### Study design

To characterise the epidemiological information held in within-host MTBC variation present in routinely generated Illumina sequence data from cultured isolates, we conducted a retrospective genomic epidemiology study in which we reanalysed sequence data from previously published MTBC household transmission studies, using household membership as a proxy for transmission linkage. We searched PubMed from database inception until Jan 31, 2024, for relevant articles published in English using the terms *“Mycobacterium tuberculosis”,* “whole genome sequencing”, “transmission”, and “household”. We selected studies for which both raw sequencing data were deposited on a public database and for which epidemiological data on household membership were additionally available. We also included a household study that focused on estimating the *M tuberculosis* substitution rate, but for which both genomic and household membership were available (Colangeli et al).^20^ We extracted information on epidemiological linkage such as household membership from the studies eligible for inclusion.

This reanalysis was considered non-human subject research by the University of Utah Institutional Review Board (IRB_00176142) and hence was exempt from full approval by the Institutional Review Board.

### Procedures

We processed raw sequence data with a previously described variant identification pipeline available on GitHub (appendix p 2). Briefly, we trimmed low-quality bases and removed adapters with Trim Galore (version 0.6.5; stringency=3).^21^ We used CutAdapt (version 4.2) to further filter reads.^22^ We used Kraken2 to taxonomically classify reads,^23^ mapped reads with *bwa* (version 0.7.15), and removed duplicates with sambamba.^24^ We called variants with GATK 4.1 HaplotypeCaller,^25^ setting sample ploidy to one, and GenotypeGVCFs. We included variant sites with a minimum depth of 5× and a minimum variant quality score of 20 and constructed consensus sequences with bcftools consensus,^26^ excluding indels. We used the R package *ape* (version 5.7) to measure pairwise differences between samples and fit a maximum likelihood tree with IQ-TREE, with 1000 ultrafast bootstrap replicates.^27,28^

### Statistical analysis

We considered minority variants as positions with two or more alleles each supported by at least 5× coverage at the same position, with the minor allele frequency above 1%, including variants across the full MTBC genome. We then quantified the proportion of minority variants occurring within PE and PPE genes. In subsequent analyses, we excluded PE and PPE genes, which might be more error prone. We compared mean per-sample minority variation found in different studies with the Wilcoxon rank sum test. We quantified the number of minority variants with different predicted variant effects, as categorised by SnpEff v.5.2.^29^ We measured associations between the total number of per-sample minority variants as well as minor allele frequency and per-sample median depth of coverage with Pearson’s correlation coefficient. For all tests, we used a significance threshold of p less than 0·05. We fit a logistic regression model for the number of per-sample minority variants including lineage, study, and sample median coverage with the base R function *glm.* We estimated odds ratios (ORs) for each covariate and characterised model uncertainty with 95% CIs.

We then measured the number of minority variants shared between household members and the number of shared minority variants between epidemiologically unrelated pairs. To assess trade-offs in sensitivity and specificity in minority variant identification, we measured shared minority variants after applying increasingly conservative minor allele thresholds: 0·5%, 1·0%, 2·0%, 5·0%, 10·0%, 20·0%, and 50·0%. We fit logistic regression models for pairwise epidemiological linkage: including (1) both genetic cluster membership (defined in different models by 12-single-nucleotide polymorphism [SNP] and five-SNP genetic distance thresholds) and shared minority variants, (2) only genetic cluster membership, and (3) only shared minority variants. We measured the performance of general linear models in classifying household pairs versus unlinked pairs with receiver operator characteristic (ROC) curves across all minor allele frequency thresholds, with the R package *yardstick* (version 1.3.1) and identified thresholds that maximised model ROC.

We tested for correlations between genetic distance between MTBC consensus sequences and shared minority variants with Pearson’s correlation coefficient. For the Colangeli et al study, which reports sampling time, we measured the association between sampling time between donor and recipient transmission pairs and number of shared minority variants with Pearson’s correlation coefficient.

Following variant identification, all analyses were conducted in R (version 4.2.2).

### Role of the funding source

The funder of the study had no role in study design, data collection, data analysis, data interpretation, or writing of the report.

## Results

We identified three household transmission studies for which both raw sequence data and epidemiological linkages were publicly available: a household transmission study in Vitória, Brazil (Colangeli et al^20^), a retrospective population-based study of paediatric tuberculosis in British Columbia, Canada (Guthrie et al^30^), and a retrospective population-based study in Oxfordshire, England (Walker et al^7^; table 1). Study design, sampling design, culture and sequencing methods, and MTBC lineage representation differed across studies (table 1, appendix p 6).

**Figure 1:**
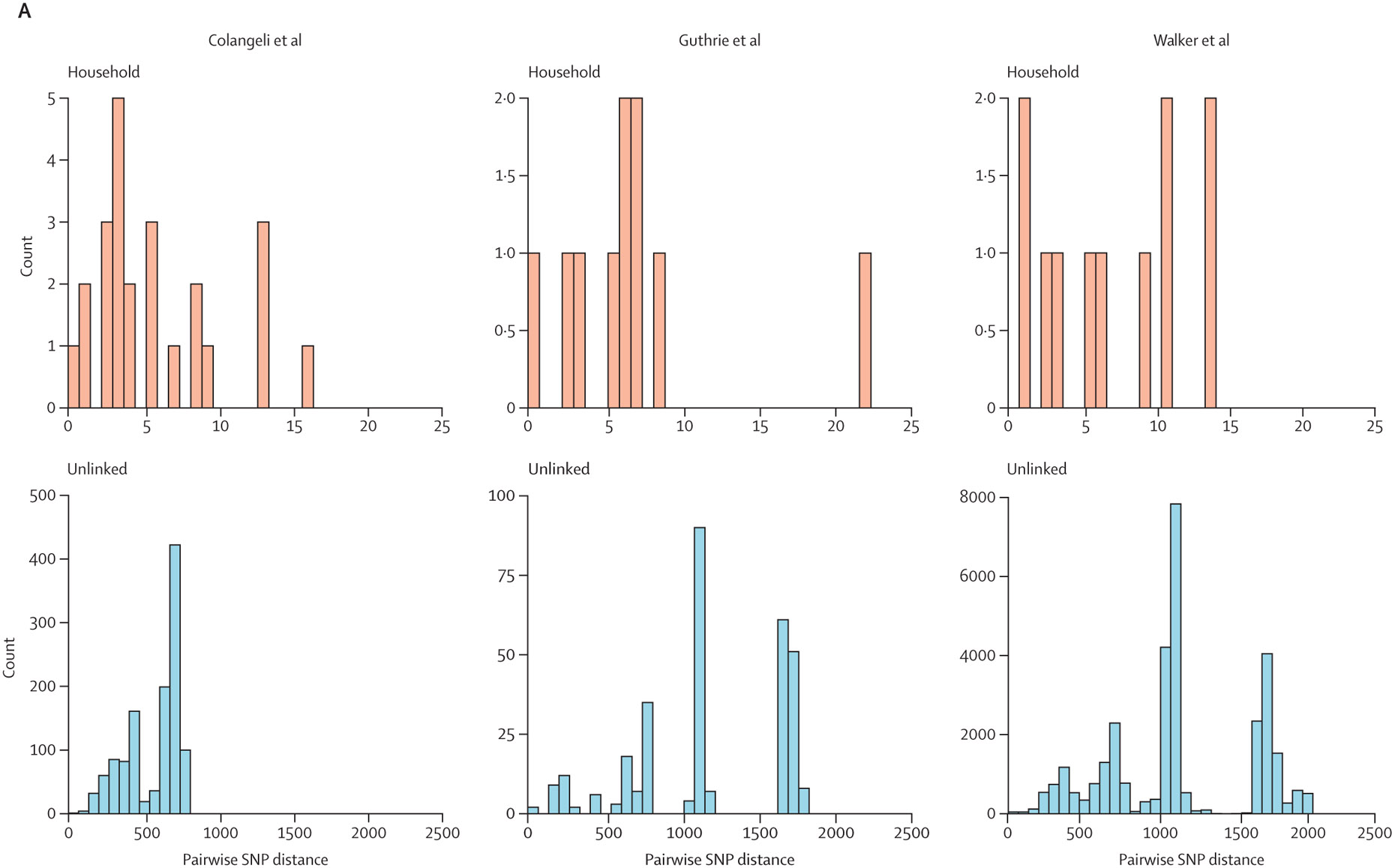

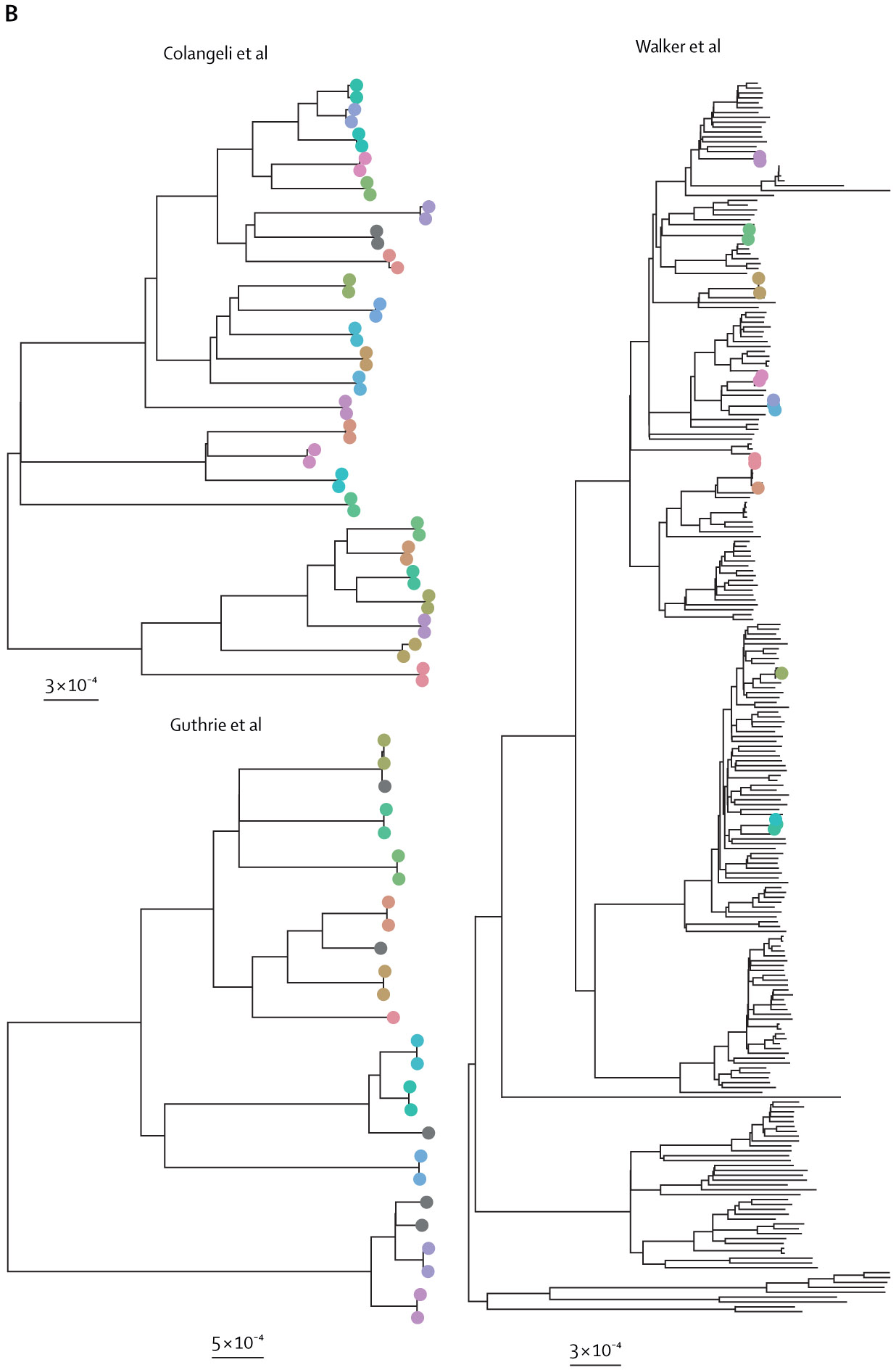
Histograms of pairwise genetic distances between MTBC consensus genomes (A), and maximum likelihood phylogeny of consensus MTBC sequences (B) (A) Histograms indicate counts of pairwise genetic distances between MTBC consensus genomes, for each included study (Colangeli at al,^30^ Guthrie et al,^30^ and Walker et al^7^) and type pairwise comparison (household and unlinked pairs). (B) Maximum likelihood phylogeny of consensus MTBC sequences for each study. Trees are midpoint rooted and tree tips are coloured by household for individuals within households or with known epidemiological links. Tree branches are in units of substitutions per site. MTBC*=Mycobacterium tuberculosis* complex. SNP=single-nucleotide polymorphism.

As reported in the original studies, we observed limited fixed variation between MTBC consensus sequences from isolates collected within the same household or among isolates from patients with epidemiological linkages compared with randomly selected pairs of sequences from the same population (figure 1A). Consensus MTBC sequences from epidemiologically linked individuals were phylogenetic nearest neighbours for each study (figure 1B). However, genetic distances between consensus sequences often exceeded the commonly used five-SNP and 12-SNP thresholds^7,32^ for classifying isolates as potentially linked in transmission, with 20 (44·4%) of 45 household pairs not meeting a five-SNP threshold and seven (15·6%) household pairs not meeting a 12-SNP threshold (figure 1A). 11 (24·4%) isolate pairs from epidemiologically linked individuals were within a genetic distance of two SNPs or less, underscoring that genomic distances alone might be limited in their resolution.

**Figure 2:**
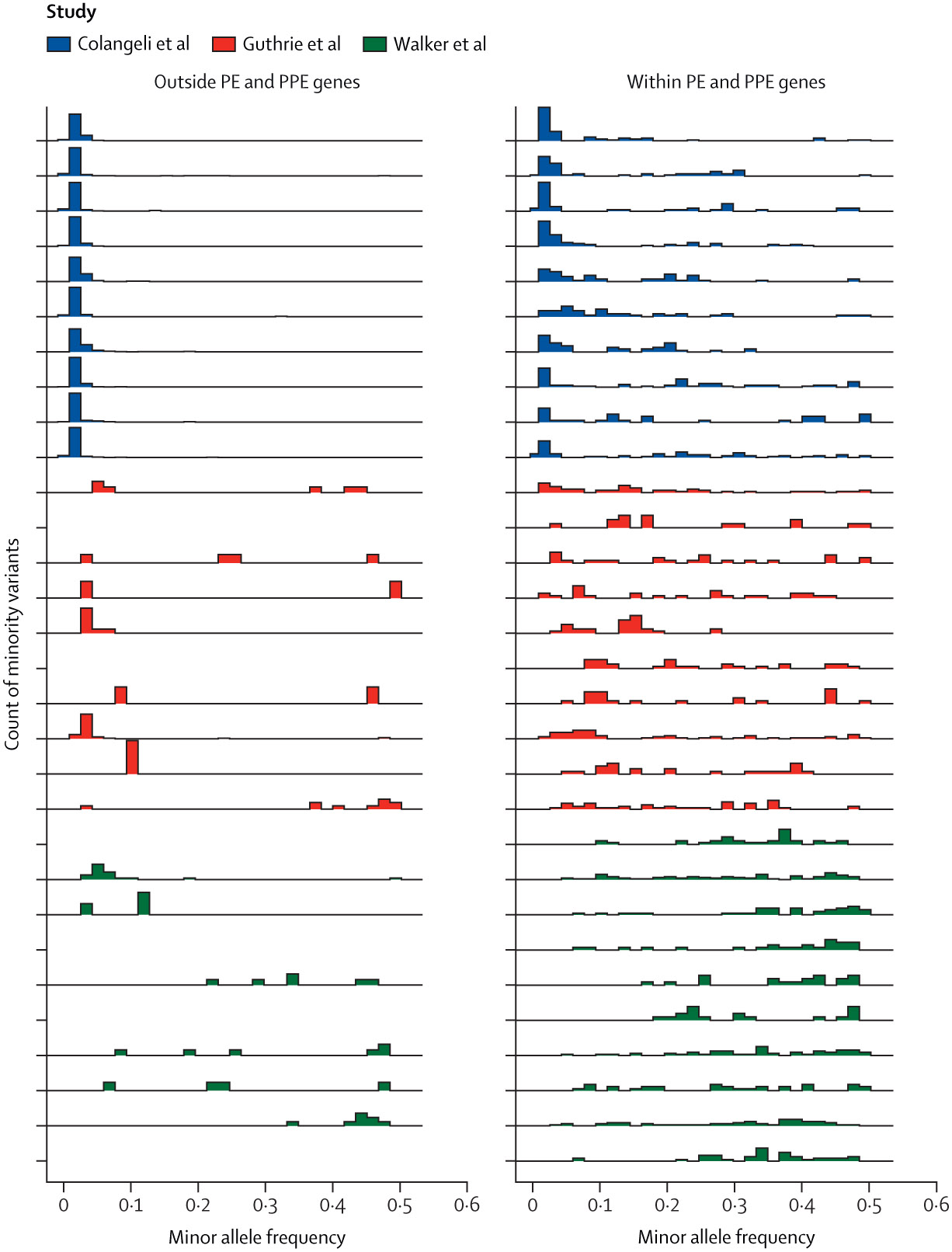
Ridgeline plot of the distribution of minority variants across minor allele frequencies for ten randomly selected samples from each study^7,20,30^ Each row indicates a unique sample and row height indicates the density of minority variants within a particular minor allele frequency bin identified for each sample, with scaling calculated separately for each panel. Panels indicate genomic region: outside PE and PPE genes and within PE and PPE genes. Some samples do not have minority variants detected outside the PE and PPE genes.

We detected a small, but measurable, minority variation above a 1% minor allele frequency threshold in routine, culture-based MTBC sequencing data, with a disproportionate number of minority variants occurring within the PE and PPE genes (mean 55·5 minority variants [24·8%] of 224·0 total minority variants in Colangeli et al; 27·1 [82·2%] of 33·0 in Guthrie et al; and 28·8 [80·1%] of 35·9 in Walker et al of all minority variants, across the studies; figure 2). Outside of the PE and PPE genes, we found significant differences in minority variation detected across studies with the Colangeli et al study (mean 168·6 minority variants [95% CI 151·4–185·9]) identifying a higher level of minority variation than both the Guthrie et al study (5·8 [1·5–10·2]; Wilcoxon rank sum test p<0·0001) and the Walker et al study (7·1 [2·4–11·9], p<0·0001; table 2). A single isolate from an individual without household contacts had evidence of two co-infecting MTBC lineages in the Walker et al study.

Most minority variants were in unique genomic locations and no minority variant was found in more than five samples in a single study (appendix p 7). 964 (50·0%) of 1929 minority variants were predicted to be missense variants and only 25 (1·3%) minority variants were stop mutations, which would generate a truncated protein. However, the five most common minority variants across all three studies occurred in intergenic regions.

Median depth of coverage was significantly correlated with the total number of minority variants detected outside the PE and PPE genes for the Walker et al study (*r*=0·15, p=0·015), whereas no association was identified in the Colangeli et al (*r*=−0·044, p=0·76) or Guthrie et al studies (*r*=−0·024, p=0·91; appendix p 8). Additionally, minor allele frequency was negatively correlated with site depth of coverage in the Colangeli et al (*r*=−0·20, p<0·0001) and the Walker et al studies (*r*=−0·31, p<0·0001), but not in the Guthrie et al study (*r*=0·11, p=0·18; appendix p 8), potentially indicating that both culture method and sequencing depth were responsible for the observed differences in recovered variation (table 1). Levels of minority variation within a sample were associated with MTBC lineage 2 isolates (OR 2·13 [95% CI 1·86–2·43]) and negatively associated with lineage 3 (0·38 [0·32–0·45]) and lineage 4 isolates (0·79 [0·69–0·90]), when compared with lineage 1 isolates, when also controlling for study and isolate median coverage.

**Figure 3:**
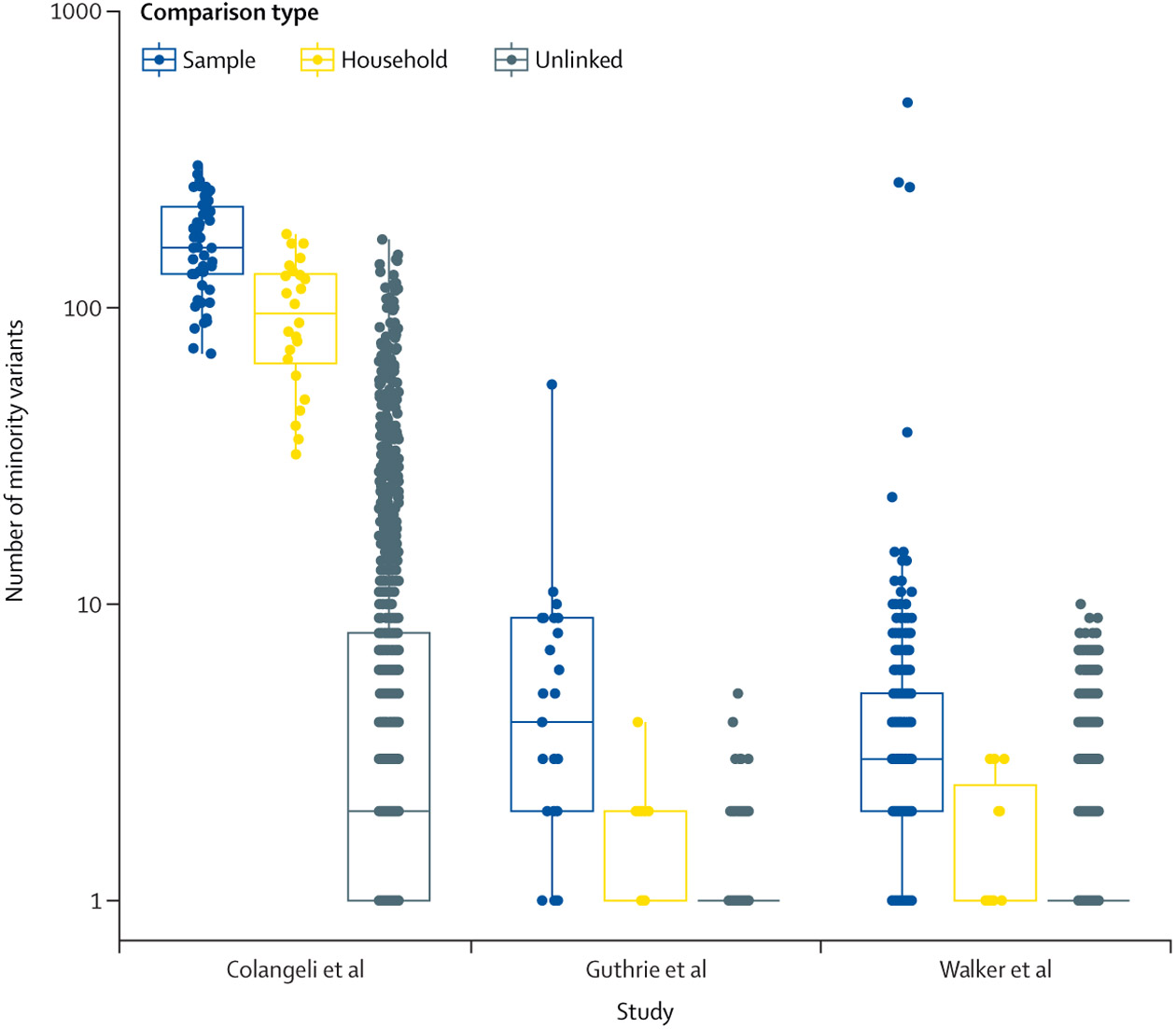
Boxplots of the number of high-quality shared minority variants between sample pairs in three previously published MTBC transmission studies^7,20,30^ with jittered points indicating pairwise observations We report the number of minority variants within samples (sample; blue), shared by household members (household; yellow), or shared by non-household members (unlinked; grey). Boxes indicate group interquartile ranges, centre lines indicate group medians, and whiskers show the range of the top and bottom 25% of values, excluding outliers. MTBC=*Mycobacterium tuberculosis* complex.

**Figure 4:**
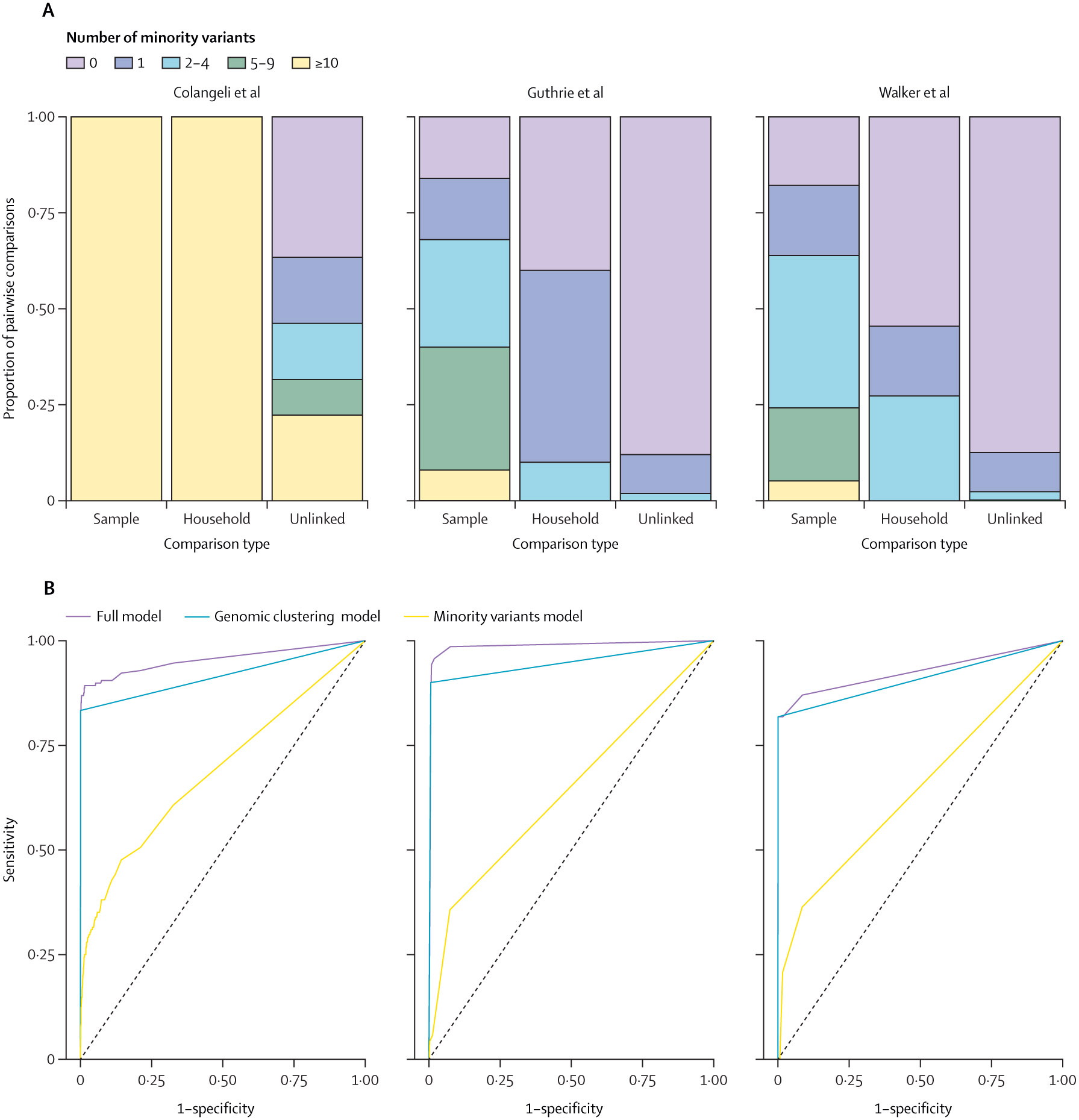
Stacked bar plots of the proportion of sample pairs across different levels of shared minority variants (A) and receiver operating characteristic curves for predicting household membership in three general linear models (B) (A) Minority variants with a minor allele frequency of 1% or more were considered. (B) Sensitivity (true positive rate) is shown as a function of 1–specificity (true negative rate). The full model includes both shared minority variants and consensus sequence-based clusters, the genomic clustering model includes the consensus sequence-based cluster only, and the minority variants model includes shared minority variants only. All models include the study as a predictor.

Isolates from household pairs shared more minority variants detected at a frequency of 1% or more and outside of PE and PPE genes than did randomly selected pairs of isolates: mean 97·7 (95% CI 79·1–116·3) shared minority variants in isolates from household pairs versus 9·8 (8·6–11·0) in isolates from randomly selected pairs in Colangeli et al; 0·8 (0·1–1·5) versus 0·2 (0·1–0·2) in Guthrie et al; and 0·7 (0·1–1·3) versus 0·2 (0·2–0·2) in Walker et al (all p<0·0001, Wilcoxon rank sum test; table 2; figure 3). This effect rapidly declined as the definition of minority variant became more stringent (appendix p 9). In each study, the distribution of shared minority variants differed significantly between epidemiologically unlinked and epidemiologically linked isolate pairs (figure 4A).

In a general linear model, shared within-host variation with a frequency of 1% or more and outside of PE and PPE genes was significantly associated with household membership (OR 1·51 [95% CI 1·30–1·71], p<0·0001) for one standard deviation increase in shared minority variants. Genomic clustering, based on a standard 12-SNP clustering distance threshold, was also significantly associated with household membership (332 [147–913], p<0·0001). When applying a five-SNP clustering distance threshold, we observed a similar association of household membership with shared minority variants (1·52 [1·38–1·67], p<0·0001). We measured the performance of general linear models in classifying household pairs versus unlinked pairs with ROC curves. Including shared within-host variation improved the accuracy of predictions in all three studies as compared with a model without within-host variation (area under the ROC curve [AUC] 0·95 *vs* 0·92 for Colangeli et al, 0·99 *vs* 0·95 for Guthrie et al, and 0·93 *vs* 0·91 for Walker et al; figure 4B). A model including within-host variation independently of consensus sequence-based clustering resulted in AUCs of 0·69 (Colangeli et al), 0·64 (Guthrie et al), and 0·64 (Walker et al; figure 4B).

To assess trade-offs in sensitivity and specificity in minority variant identification, we applied a series of increasingly conservative minor allele frequency thresholds, filtering variants detected at frequencies ranging from 0·05% to 50%. Maximum AUC for predicting household membership was 0·998 (minor allele frequency threshold: 2%) for the Colangeli et al study, 0·996 (threshold: 5%) for the Guthrie et al study, and 0·943 (threshold: 5%) for the Walker et al study (appendix p 10).

Among epidemiologically unlinked pairs, shared minority variants declined significantly with increased genetic distance between samples across all studies (appendix p 11). For household pairs, we did not find a significant correlation between the genetic distance between isolate consensus sequences and number of shared minority variants in the Colangeli et al (*r*=0·058, p=0·46) or Walker et al studies (*r*=0·18, p=0·12; appendix p 11), suggesting that this relationship might not be linear. However, we did find a positive correlation between genetic distance and shared minority variants the Guthrie et al study (*r*=0·63, p=<0·0001), which was due to a single pair with a genetic distance of greater than 20 SNPs.

Allele frequencies of shared minority variants with a frequency of 1% or more located outside of PE and PPE genes were correlated between isolates from household pairs in Colangeli et al (Pearson’s *r*=0·17, p<0·0001) and Guthrie et al (*r*=0·94, p<0·0001), but not Walker et al (appendix p 12). We predicted that sampling time might impact recovery of shared minority alleles because of changes in allele frequency between the time of sampling and time of transmission. In the Colangeli et al study, shared minority variation was negatively correlated (*r*=−0.39, p=0·058) with time between collection of isolates from household index cases and household members; however, this finding was not significant (appendix p 13). The other studies did not report sampling times.

## Discussion

To maximise the epidemiological information gleaned from the continuous evolution of MTBC, approaches to leverage biological variation more fully are needed. Here, we found that (1) within-host MTBC variation appears to persist in sequence data from culture; (2) the magnitude of within-host variation varies between and within studies and is affected by methodological choices, lineage, or both; and (3) MTBC isolates from epidemiologically linked individuals share higher levels of variation than do unlinked individuals and shared within-host variation improves predictions of epidemiological linkage. Our results suggest that minority variation could contribute epidemiological information to transmission inferences, improving inferences from consensus sequences, and that alternative approaches to culture-based sequencing might further contribute to this observed epidemiological signal.

As sequencing has become more efficient and less expensive, pathogen genomic studies have begun to describe previously uncharacterised levels of minority variation within individual hosts and shared between transmission pairs. For example, MTBC within-host variation has been used to reveal an undetected superspreader in a single large outbreak in the Canadian Arctic,^13^ shared patterns of co-infection in an outbreak in the Colombian Amazon,^33^ shared patterns of variation in a previously described compensatory mutation in Paraguay,^34^ and shared minority variants among epidemiologically linked individuals in Spain.^35^ The existence of shared minority variants suggests that variation present in a donor’s infection persists through transmission and is maintained within the recipient through population changes and immune pressures. Recently developed transmission inference approaches include pathogen within-host diversity to infer transmission events,^36^ but are not frequently applied to MTBC, which is unique in its slow substitution rate and long and variable periods of latent infection. Characterising within-host variation can also illuminate evolutionary processes—for example, parallel evolution and within-host adaptation of *Mycobacteroides abcessus* in longitudinally sampled patients.^37^

In the present study, we quantified minority variants identified by a standard variant calling pipeline indicating that new pipelines are not required to harness this level of pathogen variation. Future work is needed to develop automated, user-friendly pipelines for transmission and phylogenetic inference that include both fixed genomic differences and within-host variation.

A major challenge in pathogen genomics, including studies of within-host pathogen variation, is in distinguishing true biological variation from noise introduced by sequencing, bioinformatic analysis, or other errors. Often, pathogen genomic approaches err on the side of specificity and impose conservative variant filters. Our findings here and previously suggest that for studying transmission linkages, including low frequency minority variants could improve predictions of transmission linkage, although it is possible that some of the minority variants within individual samples and shared across samples are artifacts.^17^

Our findings underscore the further work needed to optimise approaches for highly accurate identification of both within-host and genome-wide variation. For example, because of limited variation observed in transmission clusters, there has been interest in using PE and PPE genes as an additional source of genetic variation. Our observation that minority variants are concentrated in PE and PPE genes highlights the need for testing whether long read sequencing or alternative mapping approaches can improve the accuracy of variant identification in this highly variable region.^17,19^ Work published in the past 5 years showed that pathogen enrichment approaches—through either host DNA depletion or pathogen DNA enrichment—can allow MTBC sequencing directly from clinical samples, bypassing the need for culture.^35,38^ Sequencing from positive liquid broth culture of specimens might be an intermediate step to improve detection of within-host variation.

There are several limitations to our study. First, we conducted a reanalysis of previously published sequence data from clinical MTBC samples. We therefore do not have information about the true biological variation present within samples and cannot assess sensitivity and specificity of variants identified using alternative approaches. Experiments that directly compare recovery of minority variants in known strain mixtures are required. Second, we found substantially higher within-host variation in one study (Colangeli et al^20^) than in the other two (Guthrie et al^30^ and Walker et al^7^), probably reflecting large differences in study design and sample preparation. The Colangeli et al study was prospective, and included three loops of culture for DNA extractions, whereas the Guthrie et al and Walker et al studies were retrospective and re-cultured isolates after frozen storage. The observed difference in within-host variation between studies could also reflect higher population-wide MTBC diversity circulating in a higher-incidence setting (Brazil *vs* Canada and England). Future work, including a larger number of studies, is needed to identify factors associated with recovered within-host variation—including steps in MTBC sampling, sampling time, culture, laboratory preparation, or sequencing that might have influenced recovered within-host variation. Third, we considered household transmission pairs as our reference standard for transmission linkages. Although the studies we included employed additional filters to exclude household pairs unlikely to be epidemiologically linked, these are imperfect reference standards, and it is possible that these pairs are misclassified. It is also possible that transmission outside of households resulted in undocumented epidemiological linkages. However, the impact of such mis-classifications would be to bias our results towards the null finding that shared minority variants are not more likely to be found in transmission pairs than unlinked pairs. Fourth, we do not have access to sequencing replicates of the same sputum culture or biological replicates of the same sputum to quantify the concordance of minority variants across sequencing or biological replicates. Fifth, here, we cannot differentiate between de novo variation that accumulated in culture versus variation present in the sputum, although our findings of shared variation among transmission pairs suggest that some variants were present in sputum. Finally, we took a reference-based approach to identify minority variants, which might underestimate true levels of minority variation present within individual infections and shared across infections.

Our findings of within-host variation present in cultured MTBC samples suggest that within-host MTBC variation could augment routine transmission inferences. More broadly, these finding suggest that assessing MTBC variation, including within-host variants, in addition to genome-wide variants and indels might improve both transmission and phylogenetic inferences.

## Supplementary Material

1

## Figures and Tables

**Table 1:** Characteristics of *Mycobacterium tuberculosis* complex household transmission studies

	Colangeli et al (2020)^20^	Guthrie et al (2018)^30^	Walker et al (2014)^7^
**Location**	Vitória, Brazil	British Columbia, Canada	Oxfordshire, England
**Sample size**	48 participants	26 participants	253 participants
**Confirmed household transmission pairs**	24 pairs	12 pairs*	11 pairs
**Tuberculosis incidence per 100 000 person-years** †	49·0	5·7	8·4 (reported in study)
**Study design**	Prospective household transmission study; index smear plus tuberculosis cases and household enrolled, followed up prospectively to identify secondary cases	Retrospective study; included paediatric cases aged <18 years of tuberculosis and household members	Retrospective study; all Oxfordshire residents with an *M tuberculosis* culture or clinical tuberculosis diagnosis from 2007 to 2012; tuberculosis nurses identified epidemiological linkages (ie, shared space and time)
**Culture**	Isolates cultured on Lowenstein–Jensen slants; each strain plated on Middlebrook 7H10 agar; three loops of culture were scraped and suspended in buffer	Isolates revived from frozen archival stocks on Lowenstein–Jensen slants or in MGIT liquid medium	Cultures obtained from frozen archival stocks; all cultures were grown in MGIT containing modified Middlebrooks 7H9 liquid medium and on Lowenstein–Jensen agar
**DNA extraction**	Phenol–chloroform DNA extraction	MagMA total nucleic acid isolation kit DNA extraction	Mechanical disruption with Fastprep homogeniser and Lysing Matrix B; extraction and purification with Fuji Quickgene kit
**Sequencing**	Two lanes on an Illumina HiSeq 2500	llumina HiSeqX	Illumina HiSeq
**Median sample depth**	447×	146×	103×
**NCBI Sequence Read Archive accession number**	PRJNA475130	PRJNA413593	PRJNA549270

MGIT=Mycobacteria growth indicator tube. NCBI=National Center for Biotechnology Information. *Ten pairs with high-quality sequence data available. †From the WHO 2022 country profiles^31^ unless otherwise noted.

**Table 2:** Measured within-host *Mycobacterium tuberculosis* complex variation by comparison type

	Mean (95% CI)
Colangeli et al (2020)^20^	
Sample	168·6 (151·4–185·9)
Household	97·7 (79·1–116·3)
Unlinked	9·8 (8·6–11·0)
Guthrie et al (2018)^30^	
Sample	5·8 (1·5–10·2)
Household	0·8 (0·1–1·5)
Unlinked	0·2 (0·1–0·2)
Walker et al (2014)^7^	
Sample	7·1 (2·4–11·9)
Household	0·7 (0·1–1·3)
Unlinked	0·2 (0·2–0·2)

Per-sample and shared minority variants across pairwise comparisons with different epidemiological linkages, including minority variants with allele frequency of 1% or more, outside of the PE and PPE genes, and within an expected depth.

## Data Availability

Sequence data reanalysed in this study were available from the Sequence Read Archive (accession numbers: PRJNA475130, PRJNA413593, and PRJNA549270). Accompanying metadata were accessed from the individual studies.^7,20,30^ Our variant identification pipeline and all analysis scripts for this study are available at https://github.com/ksw9/mtb-call2 and https://github.com/ksw9/mtb-within-host.

## Research in context

### Evidence before this study

We searched PubMed for articles published in English from database inception to Jan 31, 2024, using the terms “*Mycobacterium tuberculosis”,* “within-host variation” or “minority variation”, and “transmission”. We found three reviews of transmission inference and the evolutionary biology and population genomics of *Mycobacterium tuberculosis.* One genomic epidemiology study described minority variants present in a substantial proportion of isolates from a single outbreak, suggesting mixed infections. We also found studies that describe shared minority variants among epidemiologically linked individuals. However, previous studies did not quantify the added value of within-host variation to *M tuberculosis* transmission inferences or include genomic datasets from multiple studies and epidemiological settings.

### Added value of this study

We tested whether within-host variation present in routine, culture-based *M tuberculosis* whole-genome sequence data could improve inferences about transmission. To our knowledge, this is the first study to do so by applying a single bioinformatic approach to sequence data from multiple studies conducted in different epidemiological contexts. We reanalysed sequence data from three previously published household transmission studies, using household membership as a proxy for transmission linkage.

We found that household members share more within-host *M tuberculosis* variation than do epidemiologically unrelated individuals and that shared within-host variation improves predictions of household membership compared with a model that includes only fixed genetic distances between genomic sequences.

### Implications of all the available evidence

Our results show that some within-host *M tuberculosis* variation persists through culture and that within-host variation can improve transmission inferences, which are often constrained by the limited genomic variation observed in *M tuberculosis* outbreaks. The substantial differences in minority variation we observed across studies highlight the need to optimise approaches to recover and incorporate within-host variation into automated phylogenetic and transmission inferences.
