# Supplementary material for: Signatures of transmission in within-host Mycobacterium tuberculosis complex variation: a retrospective genomic epidemiology study
Source: Lancet Microbe. Author manuscript; Available in PMC 2025 Jan 29. (PMC11777664; doi:10.1016/j.lanmic.2024.06.003)

THE LANCET
Microbe

Supplementary appendix

This appendix formed part of the original submission and has been peer reviewed.
We post it as supplied by the authors.

Supplement to: Walter KS, Cohen T, Mathema B, et al. Signatures of transmission in within-host *Mycobacterium tuberculosis* complex variation: a retrospective genomic epidemiology study. *Lancet Microbe* 2025. https://doi.org/10.1016/j.lanmic.2024.06.003

Appendix

Walter et al. Signatures of transmission in within-host *Mycobacterium tuberculosis* complex variation: a retrospective genomic epidemiology study

Supplementary Methods

Genomic variation identification pipeline 2
Epidemiological data 3

Supplementary References 4

Supplementary Figures

Supplementary Figure 1. *MTBC* lineage distribution for each study 6
Supplementary Figure 2. Most observed minority variants occur at unique genomic locations 7
Supplementary Figure 3. Detection of minor alleles varies across studies 8
Supplementary Figure 4. Pairwise shared variants above alternate minor allele frequency thresholds 9
Supplementary Figure 5. Minor allele frequency threshold can alter the accuracy of predictions made with shared within-host variation 10
Supplementary Figure 6. Shared minority variants and distance between consensus sequences 11
Supplementary Figure 7. Correlation between minor allele frequencies observed in shared minority variants identified by GATK in transmission pairs 12
Supplementary Figure 8. Shared minority variants between household pairs declines within increased time between sample collection 13

Supplementary Methods

Genomic variation identification pipeline

We previously conducted a variant identification experiment to compare commonly used mapping and variant calling algorithms in *MTBC* genomic epidemiology[1]. We found that the combination of the *bwa*[2] mapping algorithm and *GATK*[3,4] variant caller routinely minimizes false positive variant calls with minimal cost to sensitivity as compared to other tool combinations[1], especially when the PE/PPE genes are excluded. We therefore used this combination of tools in our pipeline.

Briefly, we trimmed low-quality bases (Phred-scaled base quality < 20) and removed adapters with Trim Galore v. 0.6.5 (stringency=3)[5]. We used CutAdapt v.4.2 to further filter reads (--nextseq-trim=20 --minimum-length=20 --pair-filter=any)[6].To exclude potential contamination which a previous study shows can be a source of false genetic variation[7], we used Kraken2 to taxonomically classify reads and remove reads that were not assigned to the *Mycobacterium* genus or that were assigned to a *Mycobacterium* species other than *M. tuberculosis*[8]. We mapped reads with *bwa* v.0.7.15 (*bwa* mem)[2] to the H37Rv reference genome (NCBI Accession: NC_000962.3 [https://www.ncbi.nlm.nih.gov/nuccore/NC_000962.3]) and removed duplicates with sambamba[9]. We called variants with GATK 4.1 HaplotypeCaller[3], setting sample ploidy to 1, and GenotypeGVCFs, including non-variant sites in output VCF files. We included variant sites with a minimum depth of 5X and a minimum variant quality score 20 and constructed consensus sequences with bcftools consensus[10], excluding indels. We flagged SNPs in previously defined repetitive regions (PPE and PE-PGRS genes, phages, insertion sequences and repeats longer than 50 bp)[11] and excluded these variants in figures and statistics except when otherwise noted. We identified sub-lineage and evidence of mixed infection with TBProfiler v.4.2.0[12,13].

We constructed full-length consensus FASTA sequences from VCF files, setting missing genotypes to missing, and used SNP-sites to extract a multiple alignment of internal variant sites only[14]. We used the R package *ape* v.5.7 to measure pairwise differences between samples (*dist.dna*,

pairwise.deletion=TRUE)[15]. We selected a best fit substitution model with ModelFinder[16], implemented in IQ-TREE multicore version 2.2.0[17], evaluating all models that included an ascertainment bias correction for the use of an alignment of SNPs only. We then fit a maximum likelihood tree with IQ-TREE, with 1000 ultrafast bootstrap replicates[17,18] to visualize the location of household pairs in the context of study-wide variation.

We filtered variants that had coverage higher or lower than two standard deviations from the sample mean depth, reasoning that the extreme coverage was a result of incorrect mapping. We considered minority variants as positions with two or more alleles each supported by at least 5X coverage at the same position, at first, without filtering by minor allele frequency threshold.

Epidemiological data

We extracted information on household pairs from published phylogenies in the Colangeli et al. and Guthrie et al. papers. For the Walker et al. paper, household linkages were available in the data supplement (appendix p. 3).

The Colangeli et al (2020) study sequenced isolates from a prospective household transmission study[19]. To identify household transmission linkages and exclude community-acquired infection, isolates were genotyped with restriction fragment length polymorphism (RFLP) and any pairs which differed by more than one RFLP band were excluded. Additionally, household pairs infected with *M. tuberculosis* genotypes which were commonly circulating in the community (represented by an RFLP type shared by at least 10 other community isolates) were excluded.

The Guthrie et al. (2018) study genotyped *MTBC* from pediatric and adult individuals with TB in British Columbia and whole genome sequenced genotypically clustered cases. This study identified 12 household transmission linkages; of these 10 pairs had high quality sequence data available.

The Walker et al. (2014) study whole genome sequenced available isolates and TB nurses identified putative epidemiological linkages, defined as shared time and space. We considered the pairs with confirmed epidemiological linkages, and considered genomically linked, within a 12-SNP threshold,

to be transmission pairs. Transmission pairs included 9 pairs of household/family members, 1 social connection, and 1 school infection.

Supplementary References

1 Walter KS, Colijn C, Cohen T, *et al.* Genomic variant-identification methods may alter mycobacterium tuberculosis transmission inferences. *Microb Genom* 2020; **6**: 1–16.

2 Li H, Durbin R. Fast and accurate short read alignment with Burrows-Wheeler transform. *Bioinformatics* 2009; **25**: 1754–60.

3 Van der Auwera GA, O'Connor B. Genomics in the cloud : using Docker, GATK, and WDL in Terra. O'Reilly Media, 2020.

4 Van der Auwera GA, Carneiro MO, Hartl C, *et al.* From FastQ Data to High-Confidence Variant Calls: The Genome Analysis Toolkit Best Practices Pipeline. In: Current Protocols in Bioinformatics. Hoboken, NJ, USA: John Wiley & Sons, Inc., 2013: 11.10.1-11.10.33.

5 Krueger F. Trim Galore. 2019. https://github.com/FelixKrueger/TrimGalore/blob/master/Docs/Trim_Galore_User_Guide.md.

6 Martin M. Cutadapt Removes Adapter Sequences From High-Throughput Sequencing Reads. *EMBnet J* 2011; **17**. DOI:https://doi.org/10.14806/ej.17.1.200.

7 Goig GA, Blanco S, Garcia-Basteiro AL, Comas I. Contaminant DNA in bacterial sequencing experiments is a major source of false genetic variability. *BMC Biol* 2020; **18**. DOI:10.1186/S12915-020-0748-Z.

8 Wood DE, Salzberg SL. Kraken: Ultrafast metagenomic sequence classification using exact alignments. *Genome Biol* 2014; **15**: R46.

9 Tarasov A, Vilella AJ, Cuppen E, Nijman IJ, Prins P. Sambamba: fast processing of NGS alignment formats. *Bioinformatics* 2015; **31**: 2032–4.

10 Danecek P, Bonfield JK, Liddle J, *et al.* Twelve years of SAMtools and BCFtools. *Gigascience* 2021; **10**: 1–4.

11 Brites D, Loiseau C, Menardo F, *et al.* A new phylogenetic framework for the animal-adapted mycobacterium tuberculosis complex. *Front Microbiol* 2018; **9**: 2820.

12 Phelan JE, O'Sullivan DM, Machado D, *et al.* Integrating informatics tools and portable sequencing technology for rapid detection of resistance to anti-tuberculous drugs. *Genome Med* 2019; **11**: 41.

13 Coll F, McNerney R, Preston MD, *et al.* Rapid determination of anti-tuberculosis drug resistance from whole-genome sequences. *Genome Med* 2015; **7**. DOI:10.1186/s13073-015-0164-0.

14 Page AJ, Harris SR, Seemann T, *et al.* SNP-sites: rapid efficient extraction of SNPs from multi-FASTA alignments. *Microb Genom* 2016; **2**: 1–5.

15 Paradis E, Schliep K. Ape 5.0: An environment for modern phylogenetics and evolutionary analyses in R. *Bioinformatics* 2019; **35**: 526–8.

16 Kalyaanamoorthy S, Minh BQ, Wong TKF, Von Haeseler A, Jermiin LS. modelfinder: fast model selection for accurate phylogenetic estimates. 2017; **14**. DOI:10.1038/nmeth.4285.

17 Minh BQ, Schmidt HA, Chernomor O, *et al.* IQ-TREE 2: New Models and Efficient Methods for Phylogenetic Inference in the Genomic Era. *Mol Biol Evol* 2020; **37**: 1530–4.

18 Hoang DT, Chernomor O, Von Haeseler A, Minh BQ, Vinh LS. UFBoot2: Improving the Ultrafast Bootstrap Approximation. *Mol Biol Evol* 2018; **35**: 518–22.

19 Jones-López EC, Acuña-Villaorduña C, Fregona G, *et al.* Incident Mycobacterium tuberculosis infection in household contacts of infectious tuberculosis patients in Brazil. *BMC Infect Dis* 2017; **17**: 1–10.

Supplementary Figure 1. ***MTBC*** **lineage distribution for each study.** Pie chart of the lineage distribution in a) Colangeli et al. (2020), b) Guthrie et al. (2018), and c) Walker et al. (2014). Pie color indicates lineage, with pie lines indicating sublineage, to the most granular sublineage assignment made for each isolate. Sublineage names are shown for a) and b) and not for c) for ease of visualization.

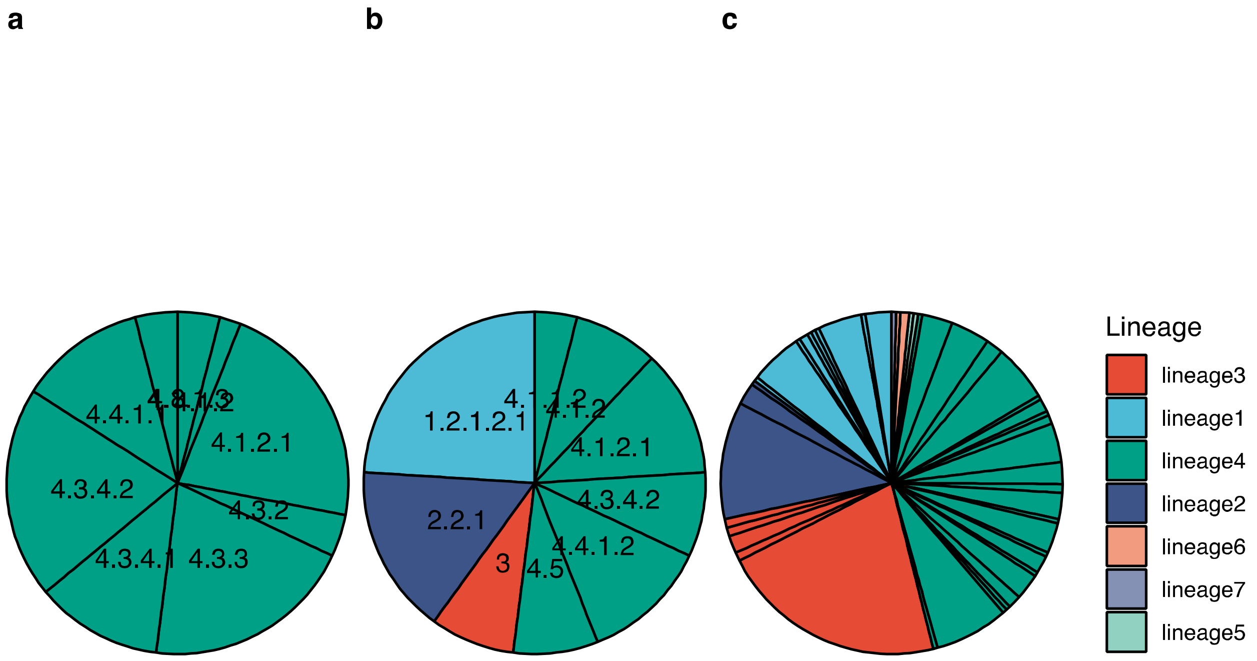

Supplementary Figure 2. Most observed minority variants occur at unique genomic locations. Histogram of the count of isolate pairs with a minority variant at a specific genomic position. Facets indicate predicted mutational effect and study. Intergenic regions, missense, and synonymous variants are included, as they comprise the majority of predicted effects of observed minority variants.

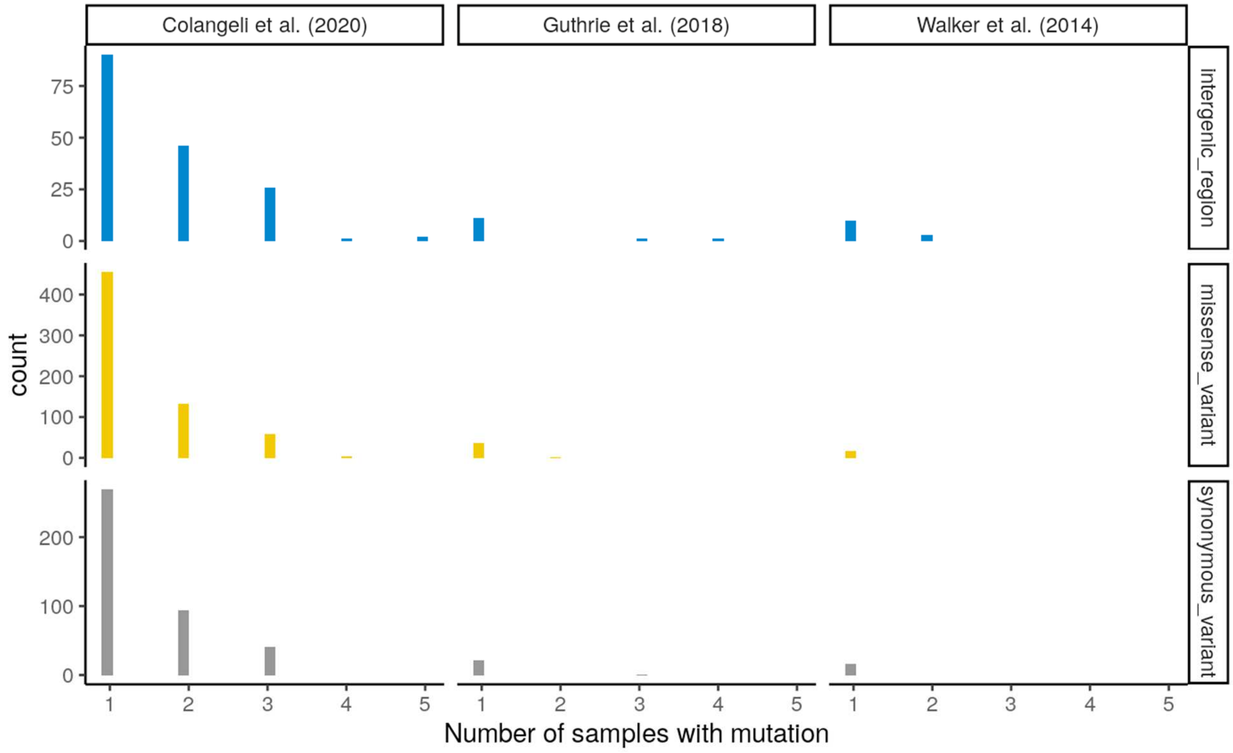

Supplementary Figure 3. Detection of minor alleles varies across studies. Scatterplots of (a) the number of minority variants above a 1% minor allele frequency threshold as a function of sample median depth of coverage and (b) minor allele frequency as a function of per-site depth, faceted by study. Pearson's correlation coefficient is reported for each study.

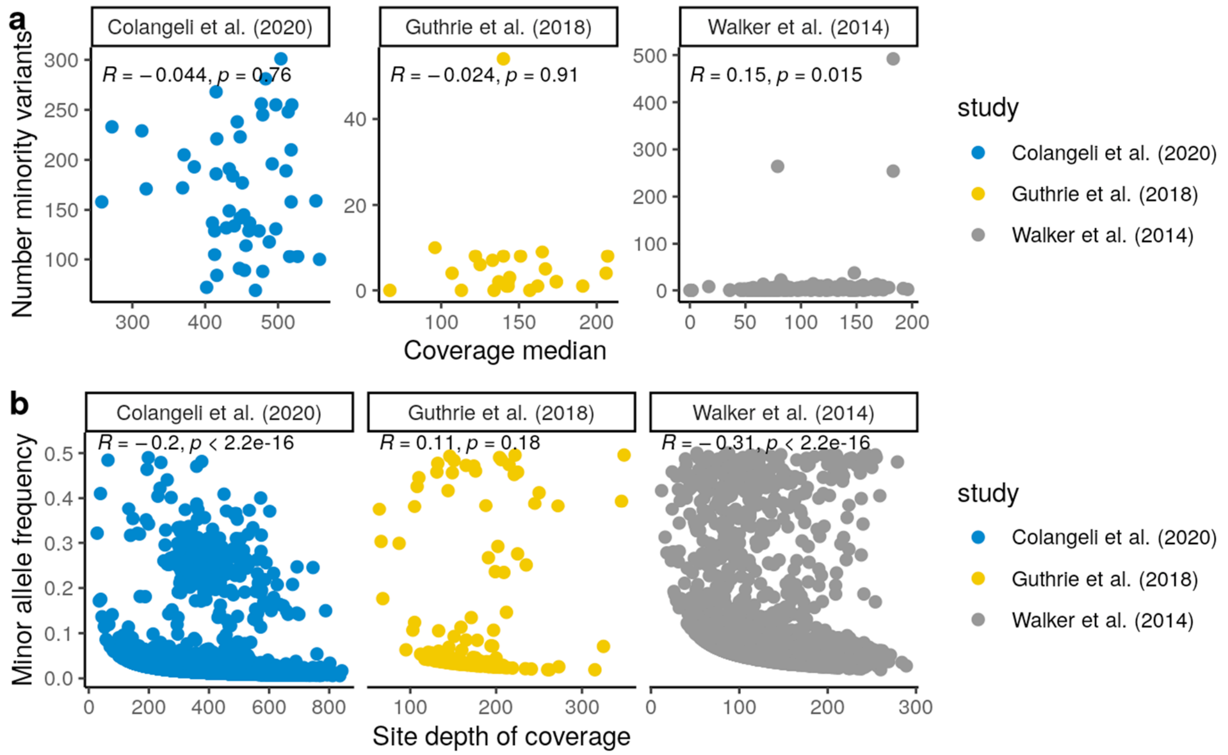

Supplementary Figure 4. Pairwise shared variants above alternate minor allele frequency thresholds. Boxplots indicate the number of shared minority variants above 1, 5, and 10% minor allele frequency thresholds. Colors indicate comparison type: sample, within-host minority variants; household, minority variants shared between household pairs; outside household, minority variants shared between individuals in different households. We report the number of minority variants within samples (“sample”, blue), shared by household members (“household”, yellow) or shared by non-household members (“unlinked”, gray). Boxes indicate group interquartile ranges and center lines indicate group medians.

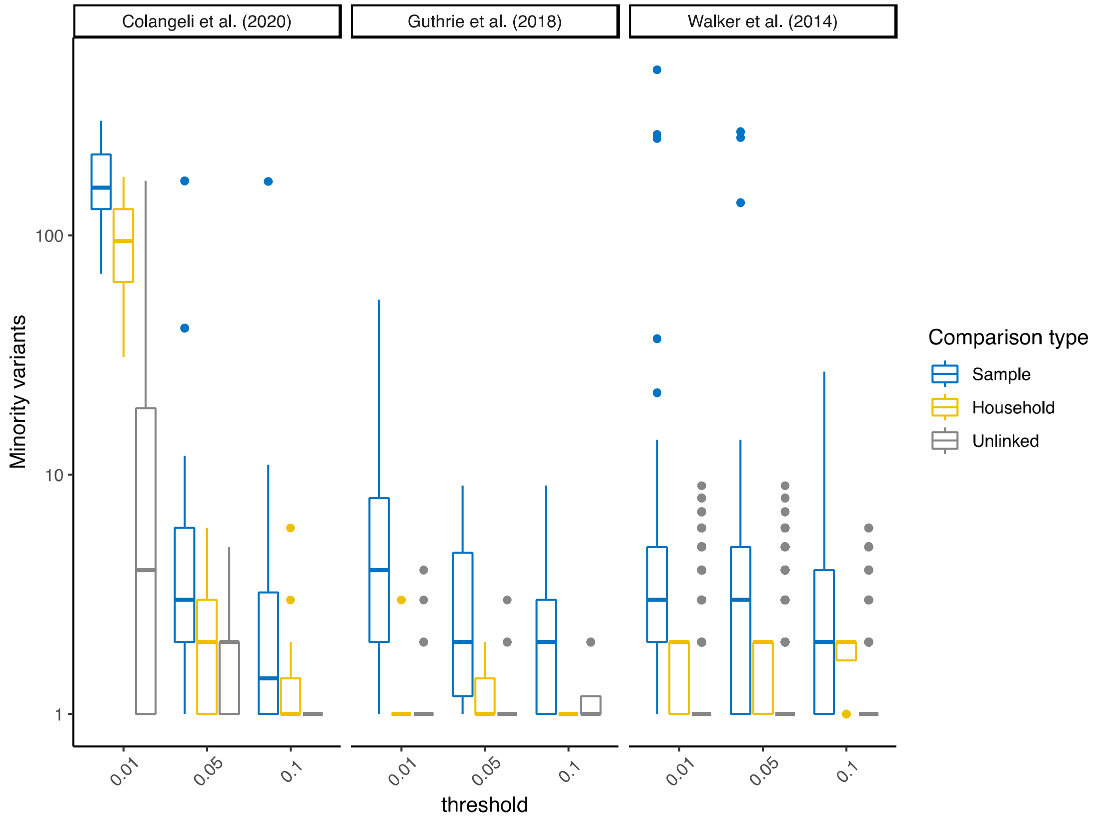

Supplementary Figure 5. Minor allele frequency threshold can alter the accuracy of predictions made with shared within-host variation. ROC curves showing sensitivity (true positive rate) as a function 1 – specificity (true negative rate) for predicting household membership in general linear models that include both shared minority variants and consensus sequence-based clusters when applying alternate minor allele frequency thresholds (color).

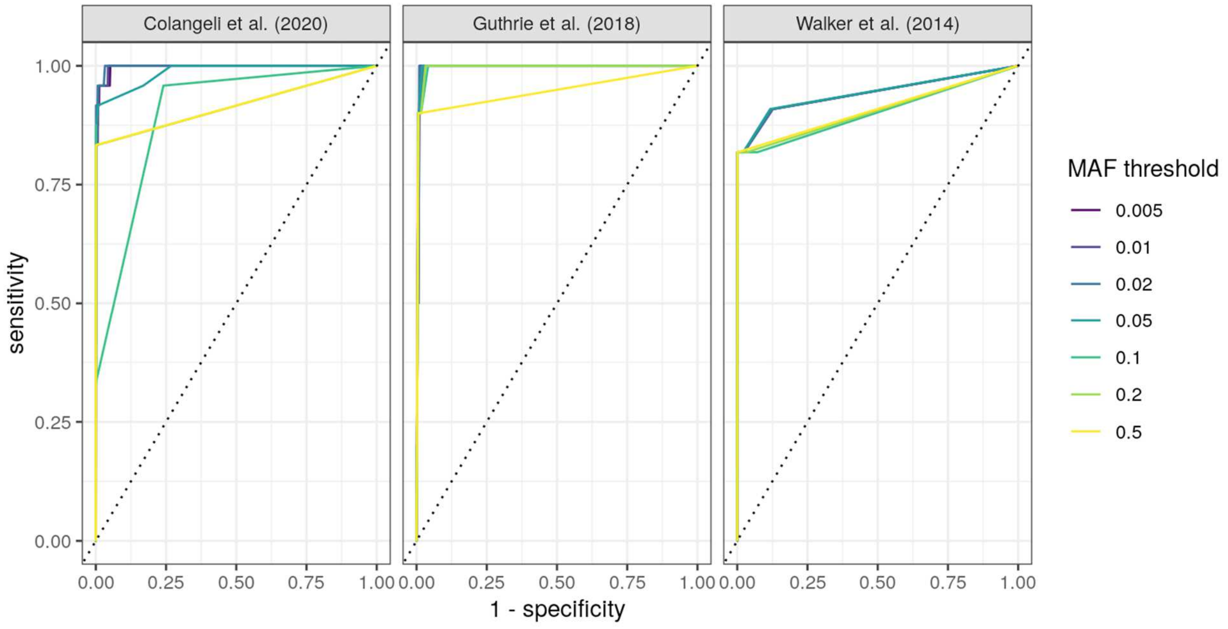

Supplementary Figure 6. Shared minority variants and distance between consensus sequences. For each pair of isolates, the number of shared minority variants versus the genetic distance between consensus sequences. Facets indicate study and comparison type and colors indicate study. Pearson's correlation coefficient is reported for each study.

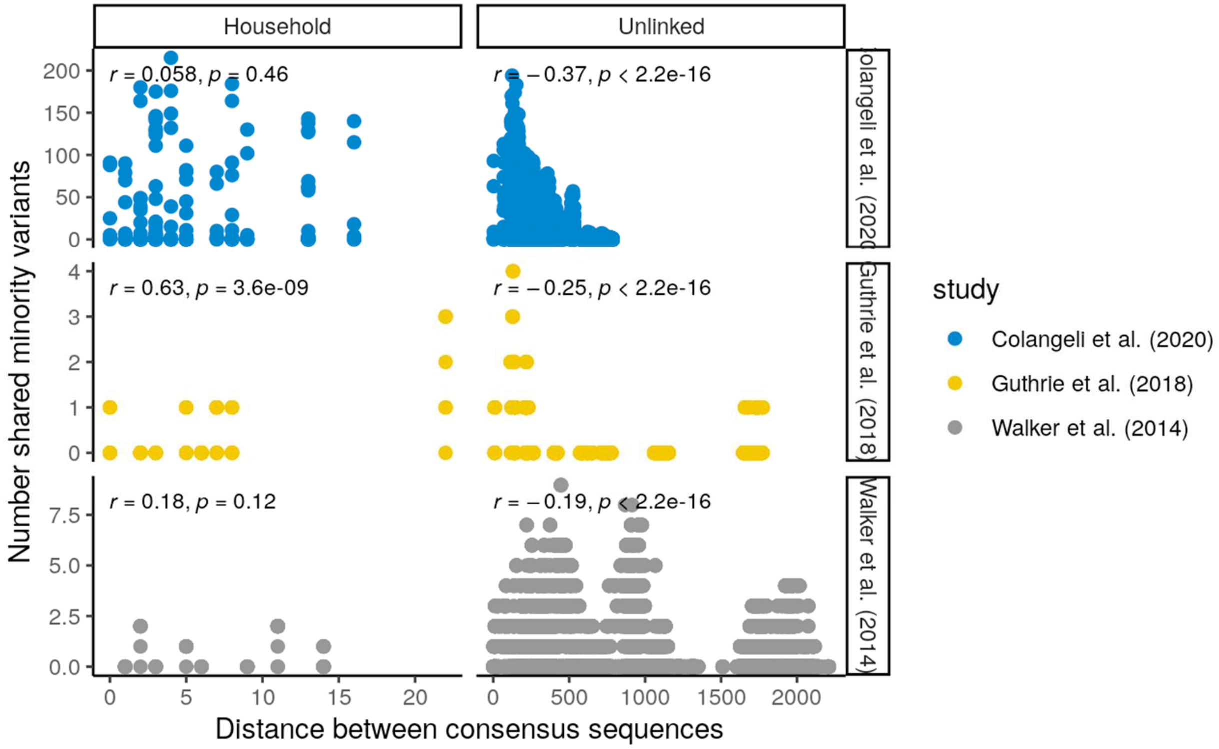

Supplementary Figure 7. Correlation between minor allele frequencies observed in shared minority variants identified by GATK in transmission pairs. Panel indicates study with Pearson's correlation coefficient and p-value for each study.

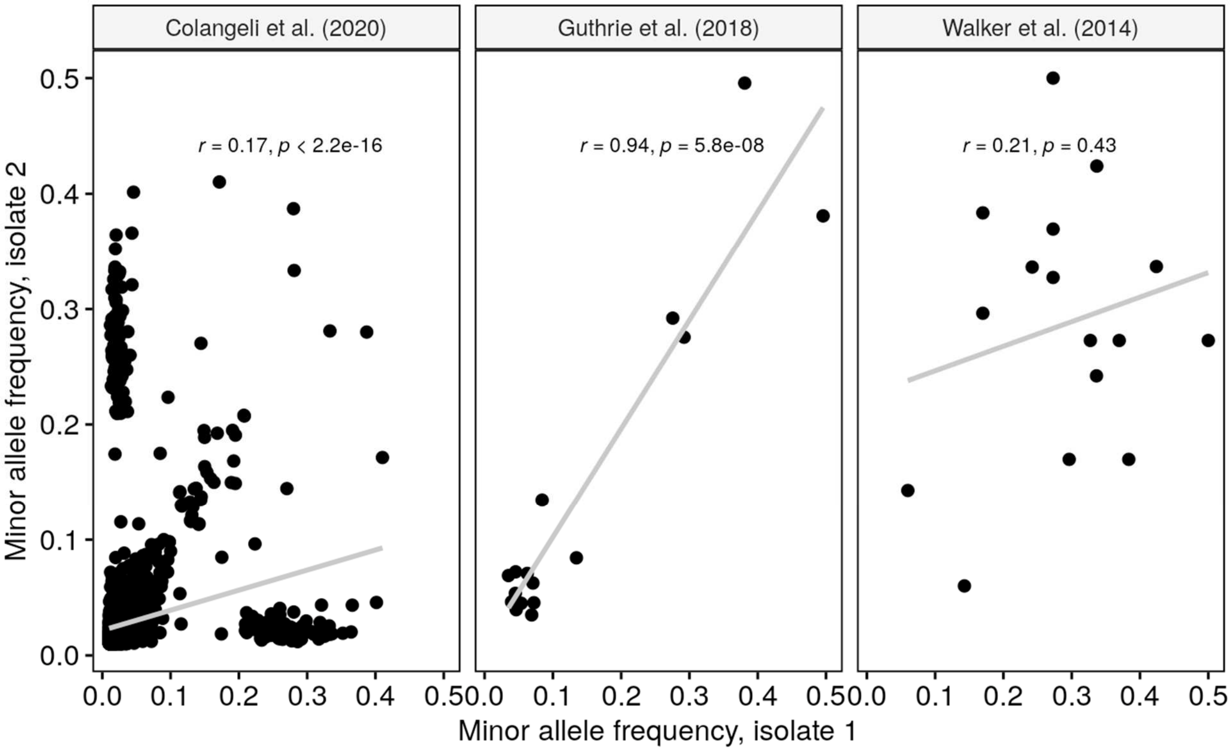

Supplementary Figure 8. Shared minority variants between household pairs declines within increased time between sample collection. The number of shared minority variants plotted against months between index and recipient host diagnosis for each transmission pair from Colangeli et al. (2020).

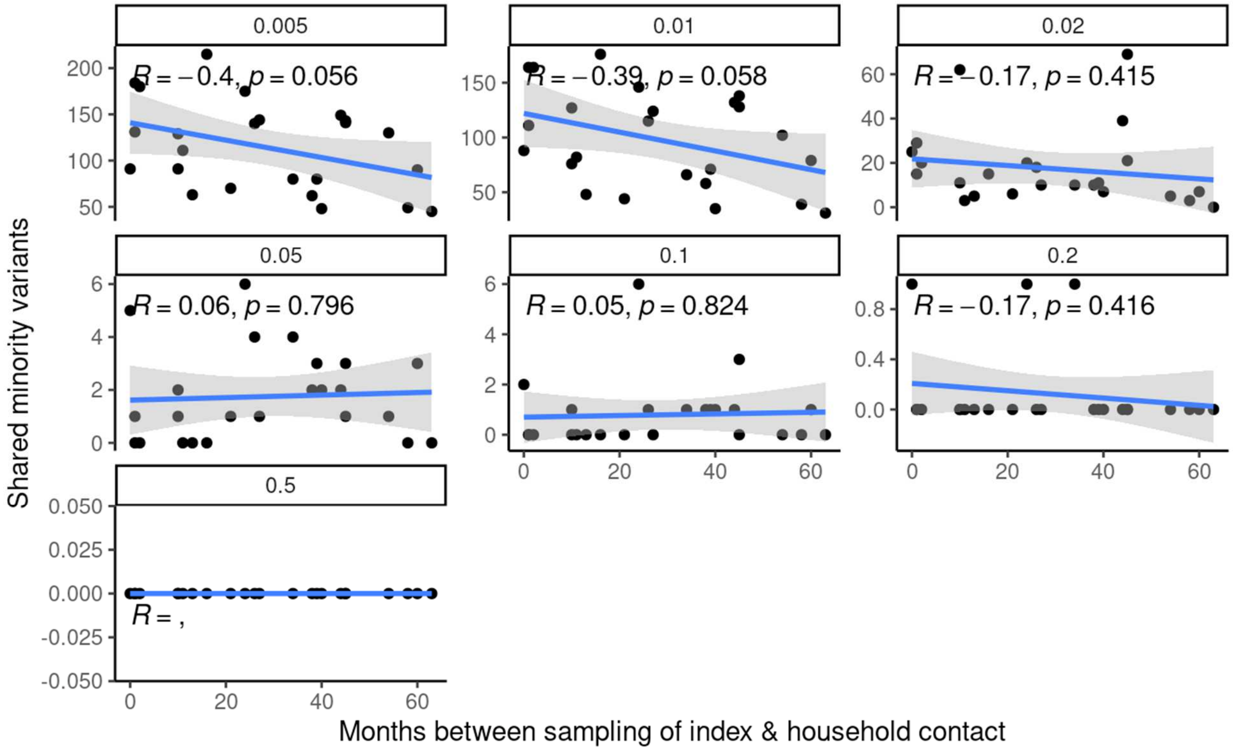

Supplement: 1 [file NIHMS2047892-supplement-1.pdf]
